# Enhancing Leadership and Management Skills in Public Health: Insights from the Public Health Management and Leadership Training Program in Uttar Pradesh, India

**DOI:** 10.2147/JHL.S484478

**Published:** 2024-12-27

**Authors:** Shalini Singh, Aman Mohan Mishra, Nishant Uppal, Rajaganapathy R, Brian Wahl, Cyrus Y Engineer

**Affiliations:** 1 Department of International Health, Johns Hopkins Bloomberg School of Public Health, Baltimore, MD, USA; 2 Uttar Pradesh Health Systems Strengthening Project, Johns Hopkins India, Lucknow, Uttar Pradesh, India; 3 Department of Human Resource Management, Indian Institute of Management, Lucknow, Uttar Pradesh, India; 4 Department of Medical Health and Family Welfare, Government of Uttar Pradesh, Lucknow, Uttar Pradesh, India

**Keywords:** public health leadership, management training, experiential learning, healthcare system strengthening, LMICs

## Abstract

**Background:**

In many Indian states, public health programs are led by clinicians without formal training in leadership and management, limiting their effectiveness. To tackle this, Uttar Pradesh’s Department of Medical, Health, and Family Welfare initiated a Public Health Management and Leadership (PHML) training program for the Level 4 (mid-career) medical officers. This program aims to enhance the leadership and management skills necessary for these officers to support them transitioning to administrative roles.

**Methods:**

The training focused on essential competencies such as leadership, communication, team building, fiscal management, and public health problem-solving. It included in-person sessions and mentored practicum, utilizing experiential learning and problem-solving group projects. Kirkpatrick’s model was used to evaluate participants’ reactions, learning outcomes, and behavior change. Feedback was analyzed using descriptive statistics across 12 training domains, while pre- and post-training test scores were compared using paired t-tests in Stata 18 to measure learning improvements. Participant interviews provided additional insights.

**Results:**

Participants reported high satisfaction with the learning environment and methods but faced challenges in applying management concepts, citing limited contextual input and faculty interaction. Learning outcomes showed moderate improvement, with average test scores rising from 53.3 to 59.6 (p = 0.003). They successfully applied a structured problem-solving framework in practicum projects and created action plans for public health challenges. Participants recommended adding topics on financing, procurement, human resources, and hospital management to support them in performing their core functions. Barriers to applying learned concepts included human resource constraints, limited autonomy, gender stereotypes, and lack of recognition.

**Conclusion:**

Emphasizing leadership competencies, experiential learning, and mentored practicum holds promise. However, customizing the curriculum to UP’s specific context, ensuring sufficient training time, focusing on core management functions, and addressing organizational barriers are vital. Integrating these recommendations into blended training that enhances core managerial skills and leadership development can strengthen workforce capabilities.

## Introduction

Health systems are dynamic, complex, and need to adapt to the ever-changing landscape of demographic, epidemiologic, and social transitions. Adapting to these challenges in resource-constrained settings necessitates highly skilled, adaptive public health leaders.^1^,^2^

Leadership and management training for program managers in Low and Middle-Income Countries (LMICs) is recommended as a key governance strategy to achieve the Universal Health Coverage.^3^ Such training programs support in developing skills for collaborative and inclusive leadership,^2^,^4^ prioritizing cross-cutting abilities to promote “systems thinking, communicating persuasively, change management, information and analytics, problem solving, and working with diverse populations”.^4–6^ Albeit few such leadership and management trainings programs are offered in LMICs,^7^ when implemented, they have positively influenced health system performance compared to settings where managers lack such training.^8^,^9^

Within India specifically, in many states, individuals trained as clinicians are often tasked to lead public health programs and hospitals without appropriate training in public health, leadership, and management. This gap in training can hinder their ability to effectively navigate the complex challenges faced by the healthcare system and implement necessary reforms. The Government of India has recognized the scarcity of appropriate skill development training as a critical issue. The 2017 National Health Policy stresses human resource development, leadership, and governance to strengthen the country’s healthcare system.^10^ It advocates for focused training programs to enhance leadership and management skills among public health professionals in decision-making roles at various levels, emphasizing competencies like strategic planning, problem-solving, communication, and stakeholder engagement.

Institutions such as the Indian Institute of Health Management Research (IIHMR), the National Institute of Health and Family Welfare (NIHFW) in New Delhi, All India Institute of Medical Sciences (AIIMS) in Delhi, the All-India Institute of Hygiene and Public Health (AIIHPH) in Kolkata and International Public Health Management Development Program by the Post Graduate Institute of Medical Education and Research, Chandigarh offer trainings on essential aspects of leadership and health management^7^. However, not all state governments have universally adopted these courses for training its public health officials; there remains scarcity of formal leadership and management training within the public health sector before assuming senior positions in the state health departments.

Uttar Pradesh(UP), India’s most populous state, with an estimated 235 million people, represents 17% of the country’s population. It is among the most socio-economically disadvantaged states, with a predominantly rural population, comprising over 76%.^11^,^12^ The state grapples with numerous public health challenges, with its health indicators often lagging behind national averages. The Maternal Mortality Ratio of 167 per 100,000 live births for UP is significantly higher than the national average of 97 per 100,000 live births; Under-five mortality rate is 32 (per 1,000 live births) for India while the same for UP is significantly high at 43 per 1,000 live births^13^ Moreover significant geographic and socio-economic inequalities are observed in the coverage of essential services.^12^ Despite a substantial rise in public health spending in UP from 2013 to 2018, government audits have revealed that the public health facilities at the primary and secondary care levels perform poorly on productivity, efficiency, service quality, and clinical care capacity.^14^ Several of these health facilities are managed by medical officers and there are governance disparities in public health system, contributing to ineffective implementation of public health policies and programs in specific districts of the state.^15^ One potential reason for this is UP lacks mandates for health workers to receive public health training, including leadership and management skills, limiting medical officers’ ability to perform essential public health functions and professionally manage public health services.^16^

Recognizing the urgency to address these gaps, the state government aims to equip its public health workforce to address these challenges through enhanced training initiatives. One such initiative, the Public Health Management and Leadership (PHML) training program started by the Government of UP(GoUP), targets mid-career or Level 4 medical officers with approximately 20 years of experience. Previous studies on public health management and leadership training programs in India have identified several issues, such as trainings lacking competencies identification for practicing public health practitioners in government health systems,^16^ being too theoretical, underutilizing institutional partnerships, or lacking clear career trajectories post-training.^17^

In response, the GoUP PHML training design was an attempt to actively address these concerns. The central idea of the training was to equip learners with leadership and management skills and their practical application rather than theoretical concepts. Additionally, there was a clear intention to build partnerships with academic institutions specializing in training for leadership, management, and public health. Furthermore, the GoUP planned to assign participants to state or district health leadership positions after the training.

By empowering mid-level public health practitioners, the program strives to drive improvements in Uttar Pradesh’s healthcare system. Equipping Level 4 medical officers with leadership and management competencies would increase their ability to perform essential public health functions, manage public health services and better govern public health facilities. In the long term this training is expected to overcome productivity, efficiency, service quality gaps within UP’s health system. We aim to document the fundamental components of the PHML training program and discuss the findings from the assessment of two pilot batches of training mid-level medical officers who participated in the capacity-building initiative. The findings from this study will inform the scale-up of the PHML training program to reach approximately 2,550 mid-level medical officers across UP. Furthermore, insights gained from this training program can serve as a valuable resource for the design and implementation of similar initiatives aimed at strengthening leadership and management competencies among public health professionals working at the state, district, and sub-district levels in other Indian states or comparable LMICs. By sharing the lessons learned and best practices from the PHML training program, this study contributes to the growing body of knowledge on effective capacity-building strategies for public health leaders in resource-constrained settings, ultimately supporting the development of resilient and responsive health systems.

## Material and Methods

### PHML Training Details

The PHML training pilot aimed to enhance mid-career medical officers’ competencies in DOMHFW-UP, preparing them to transition from clinical to administrative leadership roles and drive change in UP’s health systems.

Training was led by UP State Institute of Health and Family Welfare (SIHFW), the government agency responsible for training programs for public sector medical officers in the state. The training was launched in partnership with two academic institutions: the Indian Institute of Management Lucknow (IIML) and the Johns Hopkins Bloomberg School of Public Health (BSPH). UP SIHFW’s local expertise ensured program alignment with UP’s health landscape. IIM Lucknow in conjunction with BSPH contributed to content development based on identified competencies. Key topics included team building, organizational strategy, change management, effective communication, decision-making, and leadership. BSPH further integrated crucial public health concepts into the training, covering topics like problem-solving and analysis, partnerships, collaboration, and systems thinking in public health.

The learning objectives of the PHML training were:

1. Appreciate and recognize personal leadership style and leaderships’ role in driving teams and organizational performance.
2. Develop a range of leadership skills and behaviors including developing self, high performing teams, and organizations.
3. Lead and commit to a workplace challenge project for the mentored leadership phase of the training.

### Training Design

Adult learning theories are crucial in shaping the design and implementation of educational programs, including those for healthcare professionals. Various forms of adult learning theories have been used in past for health care professionals ranging from instrumental-behavioural, experiential, humanistic, transformative, social, motivational, reflective, and constructivist learning theories. PHML training required empowering learners to identify, reflect on their own leadership styles, challenge and change their embedded attitudes and assumptions. Therefore, training design was based on experiential learning model.^18^ Experiential learning is a form of instrumental learning where people gain knowledge and skills through hands-on experiences, reflection, forming ideas, and testing those ideas in real-world settings, creating a continuous cycle of deep learning.^18^ We also referred to the “Full Range Leadership Model(FRLM)” that covers a spectrum from low to high engagement for leading. It emphasizes that a leader can use both transactional and transformational leadership styles as needed, which, while distinct, are meant to complement each other.^19^ The select public health competencies were drawn from the core competencies to support workforce development and performance management in the Indian context identified by Bhandari et.al.^16^ The content and modules were structured in a matrix format, guiding individuals to first understand, manage, and lead themselves before advancing to managing teams and organizations.

The training curriculum wascollaboratively crafted by UP SIHFW, IIM Lucknow, and BSPH faculty, targeted skill development in various areas including self awareness, team building, leadership, communication, entrepreneurship, and innovation, fiscal management, systems thinking and problem-solving in public health. It comprised eleven structured in-person sessions and a mentored practicum. Employing experiential learning methods, sessions included a mix of didactic, participatory discussions, skill building sessions, case studies, and peer learning. Each session involved reflective exercises, surveys and peer-based feedback for learning leadership competencies. During the mentored phase, participants worked in groups to identify public health problems, devise solutions, and create resource-sensitive action plans that include activities based on available human and financial resources to optimize efficiency and effectiveness. See Supplementary File 1 for further details on the competencies and learning activities.

A total of 57 (ie, pilot batch 1 = 27 and pilot batch 2 = 30) medical officers attended the pilot training batches. To select participants, the GoUP invited nominations from all 75 districts in UP. Training participants were selected based on predefined criteria, requiring a minimum of five years of remaining service and a proven track record of excellence in clinical or responsible for public health programs.

The group had a balanced representation of districts, gender, as well as individuals involved in leading or managing public health programs and clinicians overseeing health facility administration. The participants were finally selected to represent different districts (UP is divided into 75 districts and 18 divisions). The first and second batches included participants from 32% and 24% of the state’s districts, respectively, There were 46 males and 7 females, about half of the participants held leadership positions in public health program administration or management, focusing on the implementation of national health programs at the regional and district levels. These leaders play a crucial role in the success of public health programs, overseeing large health workforces and ensuring effective delivery of public health services. The remaining participants managed clinical services at the district or block level within the UP health department. This diverse sample provided a wide range of perspectives and experiences during the training. The inclusive approach enhanced the applicability and generalizability of the results by closely representing the Level 4 doctors who were most likely to benefit from or participate in such training programs.

The PHML training was divided into three phases. The initial phase included six days of interactive in-person sessions that incorporated interactive and reflective learning activities focused on leadership and management skills. Sessions on systems thinking and problem solving were led by UP SIHFW and BSPH faculty, while IIM Lucknow conducted the remaining sessions. Participants also worked in groups to identify public health challenges in UP, which they would collectively address during the subsequent mentored practicum through a structured problem solving process. They defined the problem, identified affected populations, magnitudes, timeframes, and root causes.

During the next three-month mentored leadership phase, participants collaborated in groups of five to address problems previously identified during Phase 1. They conducted Strength, Weakness, Opportunities, Threats (SWOT) analyses, proposed managerial solutions based on a set criterion, and formulated action plans detailing responsibilities, actions, locations, timelines, and implementation methods. Mentors from UP SIHFW, IIM Lucknow, and BSPH provided guidance as necessary. Plans were reviewed by a group mentor for feedback, encouraging participants to apply new knowledge and learnings from the training.

In the final phase, a one-day post-training summit at IIM Lucknow was organized, during which participants presented their action plans from the practicum to senior DoMHFW officials. After completing the summit, participants received certificates of completion from UP SIHFW, BSPH, and IIM Lucknow.

### Training Assessment

The specific objectives of the training assessment were to understand learners’ initial feedback on their training, their perspectives regarding the training’s relevance within the UP health system, confirm their knowledge improvement, collect viewpoints on the mentored practicum, assess how learners are integrating the training inputs in their work environments and identify areas for enhancing future training based on these insights. To meet these objectives, we used the Kirkpatrick training assessment model,^20^ a standardized approach for gauging the training effectiveness of several health training initiatives. The assessment focused on the first three of the suggested four levels of training assessment. The first level, “Reaction”, assesses how much participants appreciate, engage with, and perceive the training’s relevance to their work. The second level, “Learning”, measures improvement in participants’ knowledge, gained from the training. Level 3, “Behavior”, captures participants’ reported application of the learned material in their job roles. Due to the pilot training program’s timelines and scope, it was not possible to assess comprehensively the Results or the long-term organizational impact of the training.

A mixed-methods approach was used to assess the training components. Following the completion of the six-day training, participants from both pilot batches completed a feedback survey form utilizing a 5-point Likert scale, ranging from 0 for poor to 5 for excellent. This form collected information on participants’ perspectives regarding different facets of the training, encompassing the overall design, course structure, learning materials, faculty communication, sufficiency of residential facilities, and other parameters. The feedback obtained through the training Feedback Form aided in evaluating participants’ reactions to the training.

To verify whether participants acquired the required knowledge from the training program, multiple-choice quizzes based on the training content were administered at the beginning and end of the training, scoring a maximum of 100 points. The behavior component within the Kirkpatrick model was evaluated by reviewing the action plans developed by participants at the conclusion of their three-month mentored practicum.

Additionally, six to eight weeks after the training, we conducted qualitative semi-structured, in-depth interviews with learners from both pilot batches. All 51 candidates from the two pilot training batches were eligible for the interviews and were requested to participate at the end of the six days in-person training. One investigator (AM) reached out to participants by phone or Email to schedule interviews. All participants who confirmed appointments for the virtual interview were interviewed. Interviews continued with new participants until data saturation was reached, ensuring no new themes emerged. Informed oral consent was obtained and interviews were conducted by investigators (SS) and (AM) in the local language (Hindi) via Zoom. The interviews centered on comprehensive reactions from the participants regarding the training, its relevance to their work, training application in work settings (leadership behaviors adopted after the training), experience with the mentored practicum, suggestions for improvement, scale-up strategies, and identification of any potential barriers affecting learning application. Supplementary File 2 includes the in-depth interview guide used for the interview. We achieved data saturation after interviewing 23 participants.

### Analysis

We used descriptive statistics to analyze the training feedback from the participants. Mean scores were calculated for each of the 12 training parameters across all participants, with higher scores indicating greater satisfaction in the training domain. All quantitative analyses for tracking the learning improvements based on the pre- and post-training quizzes were performed using Stata 18. We used paired t-tests to assess the statistical difference in pre- and post-training mean test scores among participants. The in-depth interviews conducted in Hindi were transcribed verbatim and translated into English. The transcripts were then coded using both deductive codes established a priori from the interview guide and inductive codes identified through a close reading of all transcripts. Following the established coding system, thematic analysis of the interview transcripts was conducted using the MaxQDA version-22 software.

### Ethics Statement

This study was determined not to be human subject research by the IRB at the Johns Hopkins Bloomberg School of Public Health (JHSPH) (FWA FWA00000287). The DoMHFW, GoUP, approved the training, and all nominated participants were informed about the training methods and assessments before the initiation of the training. In-depth interviews were conducted after training, once participants returned to their work settings. Informed oral consent was obtained before the virtual interviews, which were chosen for participants’ convenience. The consent was recorded on an audio recorder and in the investigators’ notes. Oral consent was approved by the JHSPH IRB.

## Results

Combining both the pilot training batches, 46 male and 7 female participants attended the training program. About half of these individuals occupied leadership roles in administration or program management, while the other half were overseeing or were responsible for clinical services within the UP health department. The first and second batches of training participants had representation from 32% and 24% of the districts, respectively, out of the 75 districts in UP (See Table 1).

**Table 1:** General Demographic of Participants

Characteristic		Number of Participants	%
Gender	Male	46	86.8
	Female	7	13.2
Job Category	Administrative/Programatic	27	51
	Clinical	26	49
Districts covered (out of 75)	Batch 1	24	32
	Batch 2	18	24

In this section, we describe findings from three stages of Kirkpatrick’s model: (1) reaction, (2) learning, (3) behavior. Here, we also delineate participants’ insights and recommendations for improving the training, potential scale-up strategies, and for addressing barriers affecting the application of learning.

**Figure 1:**
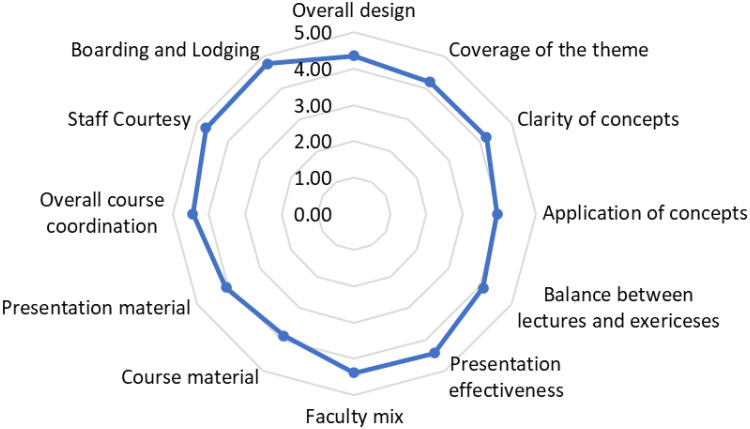
Participant Feedback Mean Scores Across 12 Training Domains.

With regard to the “reaction” domain, the satisfaction of the participants with the overall training design was high (Mean score > 4). Among the 12 training domains, participants indicated the highest satisfaction with onboarding and lodging, staff courtesy, presentation effectiveness, and course coordination (Mean scores >4.4). Conversely, mean scores were found to have been slightly low for domains related to concepts and course material clarity. See Figure 1

Participants expressed high satisfaction with the PHML training, noting it as their first systematic capacity-building experience for leadership and management. Despite prior clinical training and national program orientations, they mentioned that efforts to enhance administrative skills were limited. All participants appreciated the conducive learning environment at IIML, citing courteous faculty and engaging training methods. The training enhanced their self-awareness and realistic goal-setting skills, taught them structured problem-solving and effective communication, and emphasized team cohesion. See Table 2 on participants’ reactions to training.

**Table 2:** Participants’ Reactions to Training

Participant	Coded Themes	Quotes
**231130IDIKN**	Unique experience	“I would say that, in my opinion, this government-sponsored training initiative has been the best, and for the first time, such an excellent training has been organized in collaboration with an international institute.In my 24 years, I might have attended around three hundred and fifty training courses. Among those, this training was quite exceptional…”
**231019IDIAA**	Good learning environment	“So, this was one thing that faculty was genuinely nice and compassionate. The best part of the training was how they made us feel comfortable Physical comfort….I am talking about the residential training was comfortable Because usually when we go to different training, it is so uncomfortable passing even a day”
**231019IDIAA**	Skills to develop realistic goals and nuance to communicate pragmatic expectations	“This approach provides more flexibility and freedom to plan realistically rather than striving for unattainable targets. This training instilled confidence, allowing us to discuss and communicate realistically with our seniors, stating what is achievable this year and the next”
**231028IDIAH**	Participatory methods and peer to peer learning	“This was the second aspect I enjoyed—the company of my fellow participants and their diverse experiences in their respective fields. Some were managing as nodal officers, some as deputy CMOs, ACMOs, and so on. Getting exposure to different fields and interacting with them was very enriching”.
**231026IDIUH**	Long term goal setting and resource sensitive action plans	“The faculty discussed how to plan for even the smallest things, how to work with limited resources, and still achieve your goals. Their foresight and approach to working with limited resources impressed me greatly”
**231023IDILI**	Team cohesion	“Another important aspect is team cohesion. We learnt to multitask, segregate work effectively, delegate and understand each team member’s perspective to allocate tasks more efficiently”
**231026IDIAS**	Self -awareness	“In those five to six days of training, it was really beneficial. We learned about ourselves, our characteristics, our strengths, and weaknesses. There were exercises like identifying if we are like a turtle, eagle, or lion, and it helped us understand our qualities and where we could improve”
231026IDIAH	Structured problem solving	“It helped us understand at which levels problems are arising. We learned a methodology: create a fishbone diagram to identify causes, then solve them. All these teachings were helpful in our job, as a leadership and administrative officer”

Participants suggested increasing the training duration and incorporating more examples and case studies from the public sector to clarify concepts better. A participant shared:

“Sharing successful models or ideas from public sector contexts, or from other low-income countries would have added more value than corporate sector examples, as they don’t directly apply to our public hospital settings. We function in a wholly different atmosphere from corporate settings” *(Participant: 231026IDIHA)*

Some participants felt that allocating more time to specific topics and enhancing interaction with the faculty could have improved the clarity of concepts. As one participant suggested:

“I have a small suggestion to share. Despite having ample interaction with everyone, including the faculty members, I felt…. the interaction was slightly less. It would have been beneficial to have an activity or exclusive session during the six days, where we could all sit down with the faculty members of IIML and engage in an open conversation…….For example, if we had queries, approaching it not as a traditional teacher-student relationship but more as a peer or colleague, could have provided a different learning experience…” *(Participant: 231026IDIAH)*

We assessed learning improvement by comparing pre- and post-training test scores. Overall, quiz test scores among training participants improved by 6.26 points from an average score of 53.31 before the training to 59.56 after the training (p = 0.003). Scores improved by 8.30 points for the first batch (p = 0.001). While the test score improved by 4.29 points for the second batch, the difference was not statistically significant (p = 0.205). For those in clinical roles who attended the training, test scores also improved by 6.19 points after the training, from 54.13 to 60.31 (p = 0.010). For individuals in managerial roles, the change in test scores increased from 52.50 before the training to 58.36 after the training, but the difference of 5.86 was not statistically significant (p = 0.069).

At the behavior level, each group (Batch 1 = 5 groups and Batch 2 = 5 groups) was able to apply the structured problem-solving process learned from phase 1 and could identify gaps and root causes of their identified problems utilizing standard quality assurance tools like Ishikawa/Fishbone Diagram. At the end of the practicum, participants also proposed implementable solutions responsive to current and additional financial resources to address the identified problems. Supplementary File 3 outlines the problems recognized by ten groups from the two pilot batches and the suggested solutions.

After the three months of training, all groups presented their action plans specifying responsibilities, actions, locations, timelines, and implementation methods to the DoMHFW leadership, and faculty members from IIML and BSPH.

Participants not only applied their learning through the practicum project but could also integrate PHML knowledge into their professional contexts. Post-training, some participants shared they adopted incremental, results-driven health program management, used empathy-driven communication to motivate frontline workers, emphasized teamwork using training messages, and utilized training material to train colleagues in leadership and management communication.

“As you must know, there was Mission Indradhanush (immunization campaign) ongoing for the past three months. Many families were hesitant to take the vaccine, and numerous issues arose. So, what I did was tackle them piece by piece. I focused on the most result-oriented tasks, targeting the tougher pockets first to yield better outcomes rather than prioritizing other areas initially. Secondly, there are certain time-sensitive matters that need immediate attention, while others can be addressed later. This approach made it systematic, allowing for microplanning and gradual progress. We’re steadily moving forward….” *(Participant: 231019IDIAA)*

“Video clipping of Mandela was shown to us in the PHML training, and the faculty’s way of teaching was quite different. And after I returned, I trained my district level officers and took 3 hours lecture on what I had learnt from the PHML training. I gave them training on leadership management related communication”. *(Participant: 231130IDIAI)*

Participants discussed challenges and suggested improvements for future PHML training batches, emphasizing careful participant selection, extended and tailored training, diverse faculty, and access to data for problem-solving. They endorsed current topics but recommended additional ones for better performance in core functions like financing, procurement, human resources, and hospital management. Many stressed the importance of understanding financing dos and don’ts, attributing this to procurement fears.

Table 3 summarizes participants’ perceived challenges and suggestions for improvement.

**Table 3:** Perceived Challenges and Participants’ Recommendations for PHML Training Improvement

Training Aspect	Perceived Challenges	Suggestions for Improvement
● **Participant selection**	**●** Lack of interest and participation for some participants	**●** Deliberate participant selection based on service conduct, interest in managerial roles and PHML training
● **Training duration**	**●** Six days insufficient for in-person training	**●** Increase duration to 10–15 days. **●** Consider two rounds of training of one week each. **●** Allocate more time for faculty interaction. **●** Additional time for sessions on financing personal, professional, and social accountability, and communication.
● **Training curriculum**	**●** Corporate sector focus, examples predominantly from private sector	**●** Tailored curriculum focused on UP public health system. **●** More case studies from government health sector from India and other LMICs
● **Additional topics**	**●** Insufficient coverage of topics for routine administrative functions	**●** Include topics for **● Human resource management** – Service/cadre management rules, recruitment policies, transfer/leave policies **● Financial management**- Procurement norms, tendering process **● Hospital management**- Hospital administration, executing annual maintenance contracts on roles aligning with the hospital managers/administrators. **● Stress management techniques-**To manage day to day stressors in work settings
● **Training site and faculty mix**	**●** Low involvement of trainers from the government health system	**●** Hybrid model of training **●** SIHFW-UP as a site for scale-up. **●** Mixed pool of faculty encompassing- **●** Faculty from SIHFW-UP **●** Volunteer experts. **●** Experienced administrators **●** Management experts from IIML or other management institutions
● **Mentored practicum**	**●** Group members collaboration Delayed assignment of mentors Limited data access leading to abstract problem-solving Mismatched expectations of the training participants and faculty.	**●** Early assignment of mentors **●** Most practicum work must be covered during the in-person training. **●** Access to data, pre-identified problems **●** Allow participants to identify solutions requiring systemic changes.

**Figure 2:**
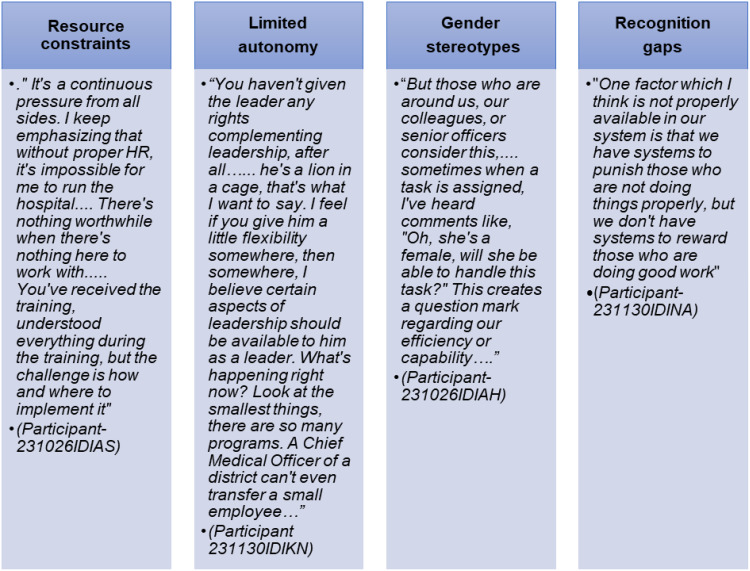
Barriers emerged that hindered their learning application.

Beyond the direct challenges of PHML training, participants also reflected on the barriers to applying what they learned, stressing the need for long-term systemic reforms in the UP health systems. Four types of barriers emerged that hindered their learning application: (1) human resource constraints, (2) limited autonomy, (3) gender stereotypes, and (4) a lack of recognition and incentives for high performers (See Figure 2).

## Discussion

Similar to other training programs,^7^,^21^,^22^ the assessment of the two pilot batches used the standard Kirkpatrick’s training evaluation framework and mixed methods approach. Analyzing the first three levels of Kirkpatrick’s model could yield valuable insights into the program. We could identify several key strengths of the PHML training.

First, the PHML training marked a shift in the training paradigm, moving away from traditional clinical capacity building or national health program orientations to exposure to disciplines mastered by institutions outside the health sector.

Second, several competencies covered in the training program were evidence-based and drawn from specific training needs of the practicing public health professionals in UP.^16^ The curriculum (Supplementary File 1) included key competencies around understanding self, communication, personal and professional accountability, ethical behavior, financial management, change management, team building promotion, cross-sectoral and stakeholder collaboration, problem-solving, and the application of systems thinking approaches. These competencies have been emphasized in other global and Indian public health leadership competency toolkits.^23–28^ Competencies for entrepreneurship, innovation, and operations management were additions incorporated by the IIML.

Third, all participants praised the learning environment and training methods, including experiential learning, participatory techniques, peer-to-peer learning, and skill-building for problem-solving. Previous studies advocate transitioning from rigid curricula to experiential methods with mentoring.^29^ Action learning, promoting collaborative problem-solving, is effective for senior leadership training.^30^ The PHML training incorporated all these approaches, including virtual group mentoring during the practicum. Post-training, participants demonstrated collaborative problem-solving, could apply learned behaviors in real-world settings, and trained their other staff on PHML competencies.

Fourth, albeit modest, PHML training improved participants’ knowledge, with scores increasing by 6.26 points from an average of 53.31 before to 59.56 after. These findings align with similar leadership training assessments in India,^31^ indicating that short residential programs effectively enhance leadership knowledge in public health professionals.

However, the PHML training assessment also revealed certain challenges related to knowledge improvement. The insufficient time allocated for specific topics contributed to a lack of clarity of concepts. Moreover, the predominant use of case studies from the private health sector may have further impeded participants’ full engagement with the training topics and hindered their thorough absorption of the material.

The assessment also uncovered unmet training needs for Level 4 medical officers, particularly in essential areas of their core managerial functions. This includes human resource administrative rules, basic human resources management, performance management, procurement, contracting processes, budgeting, and the management of public-private partnerships. Enhancing proficiency in these areas would improve Level 4 medical officers’ overall managerial capacity. These topics have been covered in other leadership trainings in India, LMICs^31^,^32^ and have also been endorsed as crucial leadership skills for health services managers by the Global Consortium for Healthcare Management Professionalization.^33^,^34^

In LMICs, health programs are decentralized, making local leaders directly accountable for services and resources.^35^ They must establish diverse partnerships, and excel in stewardship, oversight, and monitoring.^36^ However, leadership and management training in LMICs often lack these competencies, are sporadic,^37–39^ and focused on specific diseases rather than the broader skills needed for effective healthcare leadership.^36^ Our study’s insights on training strategies, competencies, learning methods, and participants’ recommendations for strengthening core health functions could serve as useful guidance for other states in India and comparable LMICs when designing leadership and management training programs for public health professionals in government roles. However, we acknowledge that these findings might be specific to the context of UP and this training program. Therefore, we recommend conducting a pilot training to validate the results before applying them to other healthcare systems.

### Recommended Training Model

Participants’ suggestions informed the creation of a scalable training model for Level 4 medical officers to be implemented by DoMHFW-UP; a two-phased blended training model was proposed.

#### Phase 1: Health Service Management and Leadership Foundation Program

Phase 1 will span six days and be conducted internally at SIHFW-UP for all Level 4 medical officers in UP. The program will include a diverse faculty from UP’s management institutions and the public health system, to cover leadership, core human resource and financial management functions, and administrative skills tailored to UP’s context. Emphasis will be on building individual leadership styles and applying proven solutions to address UP’s health systems challenges.^40^,^41^ The mentored practicum will be integrated within these six days, allowing dedicated time for problem-solving projects and mentor-trainee interactions.

#### Phase 2: Advanced Public Health Leadership Program

Phase 2, will take place at IIM-L, more intensively focusing on leadership development^40^,^41^ to address complex public health issues. It will emphasize competencies like problem-solving, change management, entrepreneurship, conflict management, and organizational leadership. Personalized mentoring and practicum projects integrated into participants’ work settings may be offered. Phase 2 will occur six months after Phase 1, targeting a select group of Level 4 medical officers. These candidates will be identified using a “composite performance index” based on Phase 1 knowledge scores and colleague feedback. A stringent selection process will ensure only motivated candidates participate, addressing disengagement concerns highlighted from the pilot batches’ assessment.

### Overcoming Barriers to Learning Application

Participants emphasized the challenge of limited autonomy and resource constraints in public sector leadership, which has been noted in other related literature to the public sector.^42^,^43^ These issues highlight the need for concurrent focus on existing systemic reforms alongside training initiatives, to enable a holistic leadership performance. Peer collaboration and an integrated leadership approach, blending transactional and transformational behaviors, are other suggested strategies to support leaders in resource-limited settings and can be tried to nurture public health leaders in UP. Transactional leadership manages immediate issues with defined processes, while transformational leadership encourages experimentation and challenges the status quo.^44^ Measures tackling gender stereotypes, adapting organizational systems to support women leaders, and financial or non-financial incentives to reward high-performing leaders also look necessary to obtain full gains from the leadership training program at UP.

## Limitations

While this study was useful in assessing immediate learning outcomes and identifying recommendations for strengthening the PHML training, it could not analyze Level 4 Results from Kirkpatrick’s model that determine if the training could achieve broader, longer-term organizational goals. Measuring the impact of training on Level 4 is complex due to difficulties in establishing causal links. Using a 360-degree feedback evaluation as a proxy for long-term results has been successful in other settings.^22^ Future studies can evaluate the proposed new training model’s long-term impact by combining the four levels model with experimental research design. Additionally, studies on assessing leadership training programs underscore the significance of cultural factors both within and outside organizations. As we learnt from this study itself that participants identified barriers to applying their learning that are closely intertwined with the region’s cultural context. In a hierarchical culture, limited support and resources restrict autonomy, leading to participant frustration and diminished training effectiveness. Gender stereotypes also limit women participants’ opportunities to apply their learning, hindering inclusive leadership development. Furthermore, in a context that does not rewards meritocracy, individuals may feel their innovative efforts are undervalued, which discourages engagement and motivation to effectively apply training insights. Therefore, using a cultural perspective to evaluate the proposed leadership development program model in UP could also deepen insights into how culture, context, and situations influence leadership effectiveness. This approach has the potential to provide valuable insights for advancing both research and practice of leadership development.^45^

## Conclusion

The evaluation of the PHML training program in UP offers insights into enhancing leadership and management among mid-level public health professionals. The focus on leadership competencies, experiential learning, and mentored practicum shows promise in improving skills. However, customizing the curriculum to UP’s context, ensuring ample time for key topics, and addressing learning barriers in the organizational environment are crucial. Integrating these recommendations into a scalable, blended training model can strengthen the Department of Medical Health and Family Welfare’s efforts in enhancing public health workforce capabilities and foster a more resilient health system. Additionally, assessing the long-term impact and broader public health implications of this training with feedback for improvements will provide a more comprehensive perspective on the program’s contributions.

## References

[1] Frenk J, Chen L, Bhutta ZA. et al. Health professionals for a new century: transforming education to strengthen health systems in an interdependent world. *Lancet*. 2010;376(9756):1923–1958. doi:10.1016/S0140-6736(10)61854-5 21112623

[2] Brandert K, Alperin M, Lloyd LM, et al. Learning to Lead: 3 Models to Support Public Health Leadership Development. *J Public Health ManagePract*. 2022;28(Supplement 5):S203. doi:10.1097/PHH.0000000000001519 35867490

[3] WHO. Health systems governance for universal health coverage action plan: department of health systems governance and financing. Accessed December 23, 2023. https://www.who.int/publications-detail-redirect/WHO-HSS-HSF-2014.01.

[4] Kaufman NJ, Castrucci BC, Pearsol J, et al. Thinking Beyond the Silos: emerging Priorities in Workforce Development for State and Local Government Public Health Agencies. *J Public Health Manag Pract*. 2014;20(6):557–565. doi:10.1097/PHH.0000000000000076 24667228 PMC4207571

[5] Czabanowska K, Rethmeier KA, Lueddeke G, et al. Public health in the 21st century: working differently means leading and learning differently. *Eur J Public Health*. 2014;24(6):1047–1052. doi:10.1093/eurpub/cku043 24709511

[6] Fraser M, Castrucci B, Harper E. Public Health Leadership and Management in the Era of Public Health 3.0. *J Public Health ManagePract*. 2017;23(1):90. doi:10.1097/PHH.0000000000000527 27870719

[7] Upadhyay K, Goel S, John P. Developing a capacity building training model for public health managers of low and middle income countries. *PLoS One*. 2023;18(4):e0272793. doi:10.1371/journal.pone.0272793 37083569 PMC10121058

[8] Chelagat T, Rice J, Onyango J, Kokwaro G. An Assessment of Impact of Leadership Training on Health System Performance in Selected Counties in Kenya. *Front Public Health*. 2021;8:550796. doi:10.3389/fpubh.2020.550796 33732670 PMC7956995

[9] Martineau T, Raven J, Aikins M, et al. Strengthening health district management competencies in Ghana, Tanzania and Uganda: lessons from using action research to improve health workforce performance. *BMJ Global Health*. 2018;3(2):e000619. doi:10.1136/bmjgh-2017-000619 PMC5898347 29662692

[10] Ministry of Health and Family Welfare, Government of India, National Health Policy, PDF 2017. Accessed December 25, 2023. Available from: https://main.mohfw.gov.in/sites/default/files/9147562941489753121.pdf.

[11] Mohfw. Population Projection Report 2011-2036.pdf, Census of India, Ministry of Health and Family Welfare, Government of India July 2020. Accessed October 26, 2024. Available from: https://mohfw.gov.in/sites/default/files/Population%20Projection%20Report%202011-2036%20-%20upload_compressed_0.pdf.

[12] Halli SS, Alam MT, Namasivayam V, et al. Geographic and socioeconomic inequalities in the coverage of contraception in Uttar Pradesh, India. *Reproductive Health*. 2024;21(1):50. doi:10.1186/s12978-024-01784-3 38600560 PMC11007924

[13] Epariyojana, Uttar Pradesh MoSPI National Indicator Framework Progress Report, Government of Uttar Pradesh 2023. Accessed October 26, 2024. Available from: https://epariyojana.up.gov.in/sdg/Dash_cif/FINAL/State%20Report%20&%20Publication/UP%20Progress%20Report%20based%20on%20NIF/NIF%20progress%20report_2023.pdf.

[14] CAG. Performance Audit of the Comptroller and Auditor General of India on Report_No_2_of_2019_Hospital_Management_in_Uttar_Pradesh_Government_of_Uttar_Pradesh.pdf. Accessed October 26, 2024. Available from: https://cag.gov.in/webroot/uploads/download_audit_report/2019/Report_No_2_of_2019_Hospital_Management_in_Uttar_Pradesh_Government_of_Uttar_Pradesh.pdf.

[15] Anand M. Health Status and Health Care Services in Uttar Pradesh and Bihar: a Comparative Study. *Ind J Public Health*. 2014;58(3):174. doi:10.4103/0019-557X.138624 25116823

[16] Bhandari S, Wahl B, Bennett S, Engineer CY, Pandey P, Peters DH. Identifying core competencies for practicing public health professionals: results from a Delphi exercise in Uttar Pradesh, India. *BMC Public Health*. 2020;20(1):1737. doi:10.1186/s12889-020-09711-4 33203407 PMC7670983

[17] Miller E, Reddy M, Banerjee P, et al. Strengthening institutions for public health education: results of an SWOT analysis from India to inform global best practices. *Human Resources for Health*. 2022;20(1):19. doi:10.1186/s12960-022-00714-3 35183208 PMC8857736

[18] Mukhalalati BA, Taylor A. Adult Learning Theories in Context: a Quick Guide for Healthcare Professional Educators. *J Med Educ Curr Develop*. 2019;6:2382120519840332. doi:10.1177/2382120519840332 PMC6458658 31008257

[19] Lindberg C The Full Range Leadership Model. Leadership Ahoy! 2021. Accessed November 2, 2024. https://www.leadershipahoy.com/full-range-leadership-model/.

[20] Kurt DS. Kirkpatrick Model: four Levels of Learning Evaluation. Educational Technology. 2016. Accessed April 27, 2024. https://educationaltechnology.net/kirkpatrick-model-four-levels-learning-evaluation/.

[21] MacVarish K, Kenefick H, Fidler A, Cohen B, Orellana Y, Todd K. Building Professionalism Through Management Training: new England Public Health Training Center’s Low-Cost, High-Impact Model. *J Public Health Manag Pract*. 2018;24(5):479–486. doi:10.1097/PHH.0000000000000693 28991053 PMC6078487

[22] Steensma H, Groeneveld K. Evaluating a training using the “four levels model. *J Workplace Learning*. 2010;22(5):319–331. doi:10.1108/13665621011053226

[23] American College of Health Care Executives. *Competencies Assessment Tool PDF 2020*. 2020.

[24] National Health Services Leadership Academy. *Leadershipframework-Summary*. 2011.

[25] Strudsholm T, Vollman AR. Public health leadership: competencies to guide practice. *Healthc Manage Forum*. 2021;34(6):340–345. doi:10.1177/08404704211032710 34601957 PMC8547231

[26] Empowering Health Leadership. Empowering Health Leadership. Accessed March 2, 2024. Avaialble from: https://www.empoweringhealthleadership.com.

[27] Report-on-public-health-leadership-training-Odisha-Faculity of Public Health, UK and State Institute of Health and Family Welfare, Odisha March-2017.pdf.

[28] Developing leadership and management competencies in low and middle-income country health systems: a review of the literature; Working paper, Resilient and Responsive Health Systems, April 2014. Accessed December 23, 2023. Available from: https://assets.publishing.service.gov.uk/media/57a089d6ed915d622c000415/WP4_resyst.pdf.

[29] Rodríguez DC, Jessani NS, Zunt J, et al. Experiential Learning and Mentorship in Global Health Leadership Programs: capturing Lessons from Across the Globe. *Ann Glob Health*. 2021;87(1):61. doi:10.5334/aogh.3194 34307064 PMC8284496

[30] Rasa J. Developing effective health leaders: the critical elements for success. *J Hosp Manage Health Pol*. 2020;4:6. doi:10.21037/jhmhp.2019.11.02

[31] Gulati K, Singh AR, Kumar S, Verma V, Gupta SK, Sarkar C. Impact of a leadership development programme for physicians in India. *Leadersh Health Serv*. 2019;33(1):73–84. doi:10.1108/LHS-05-2019-0027

[32] Jiyenze MK, Sirili N, Ngocho JS, et al. Strengthening health management, leadership, and governance capacities: what are the actual training needs in Tanzania? *Health Sci Rep*. 2023;6(3):e1158. doi:10.1002/hsr2.1158 36949870 PMC10027058

[33] International Hospital Federation. *Leadership_Competencies for Healthcare_Services_Managers by Global Consortium for Healthcare Management Professionalization*. 2015.

[34] Hahn CA, Gil Lapetra M. Development and Use of the Leadership Competencies for Healthcare Services Managers Assessment. *Front Public Health*. 2019;7:421978. doi:10.3389/fpubh.2019.00034 PMC6403121 30873397

[35] WHO_HSS_healthsystems -TOWARDS BETTER LEADERSHIP AND MANAGEMENT IN HEALTH: REPORT ON AN INTERNATIONAL CONSULTATION ON STRENGTHENING LEADERSHIP AND MANAGEMENT IN LOW-INCOME COUNTRIES 29 January - 1 February 2007 Accra, Ghana2007.10_eng.pdf. Accessed December 25, 2023. https://iris.who.int/bitstream/handle/10665/70023/WHO_HSS_healthsystems_2007.10_eng.pdf?sequence=1&isAllowed=y.

[36] Edmonstone J. Leadership development in health care in low and middle-income countries: is there another way? *Int J Health Plan Manage*. 2018;33(4):e1193–e1199. doi:10.1002/hpm.2606 30052279

[37] Jones DS, Tshimanga M, Woelk G, et al. Increasing leadership capacity for HIV/AIDS programmes by strengthening public health epidemiology and management training in Zimbabwe. *Hum Resour Health*. 2009;7(1):69. doi:10.1186/1478-4491-7-69 19664268 PMC2731729

[38] Muhimpundu MA, Joseph KT, Husain MJ, et al. Road map for leadership and management in public health: a case study on noncommunicable diseases program managers’ training in Rwanda. *Int J Health Promot Educ*. 2018;57(2):82–97. doi:10.1080/14635240.2018.1552178 33173440 PMC7651004

[39] Alkhawaldeh JM, Soh KL, Mukhtar F, et al. Stress management training program for stress reduction and coping improvement in public health nurses: a randomized controlled trial. *J Adv Nurs*. 2020;76(11):3123–3135. doi:10.1111/jan.14506 32856353

[40] Day DV. Leadership development:: a review in context. *Leadersh Q*. 2000;11(4):581–613. doi:10.1016/S1048-9843(00)00061-8

[41] Day D, Bastardoz N, Bisbey T, Reyes D, Salas E. Unlocking Human Potential through Leadership Training & Development Initiatives. *Behav Sci*. 2021;7(1):1.

[42] Seidle B, Fernandez S, Perry JL. Do Leadership Training and Development Make a Difference in the Public Sector? A Panel Study. *Public Administration Rev*. 2016;76(4):603–613. doi:10.1111/puar.12531

[43] Ferguson J, Ronayne P, Rybacki M. Public Sector Leadership Challenges. *Center for Creative Leadership*. 2014:1–2.

[44] Baškarada S, Watson J, Cromarty J. Balancing transactional and transformational leadership. *Intl J Orgl Analysis*. 2017;25(3):506–515. doi:10.1108/IJOA-02-2016-0978

[45] Edwards G, Sharon T. A Cultural Approach to Evaluating Leadership Development. Accessed June 19, 2024. https://www.researchgate.net/publication/258123689_A_Cultural_Approach_to_Evaluating_Leadership_Development.

